# A Comprehensive Review with Future Prospects on the Medicinal Properties and Biological Activities of *Curcuma caesia* Roxb.

**DOI:** 10.1155/2023/7006565

**Published:** 2023-01-17

**Authors:** Nurul Najiha Ain Ibrahim, Wan Aida Wan Mustapha, Noor-Soffalina Sofian-Seng, Seng Joe Lim, Noorul Syuhada Mohd Razali, Arnida Hani Teh, Hafeedza Abdul Rahman, Ahmed Mediani

**Affiliations:** ^1^Department of Food Sciences, Faculty of Science and Technology, Universiti Kebangsaan Malaysia, 43600 UKM Bangi, Selangor, Malaysia; ^2^Centre of Excellence, Innovation Centre for Confectionery Technology (MANIS), Faculty of Science and Technology, Universiti Kebangsaan Malaysia, 43600 UKM Bangi, Selangor, Malaysia; ^3^Institute of Systems Biology (INBIOSIS), Universiti Kebangsaan Malaysia, 43600 UKM Bangi, Selangor, Malaysia

## Abstract

Plants are the primary source of the food chain and are rich in nutrients and biochemical compounds that mainly give beneficial effects to humans as well as other living organisms. *Curcuma caesia* Roxb. is a family member of *Zingiberaceae* commonly known as black turmeric. The leaves and rhizomes of this plant are extensively used in Ayurvedic medicine and as traditional remedies for various ailments. The aromatic rhizomes and leaves are due to the presence of essential oils reported as camphor, ar-turmerone, (Z)-*β*-ocimene, ar-curcumene, 1,8-cineole, *β*-elemene, borneol, bornyl acetate, tropolone, ledol, *β*-elemenone, and *α*-bulnesene. Previous research studies have revealed most of the biological activities of *C*. *caesia*, such as antioxidant, antimicrobial, and anti-inflammatory properties, which are due to the presence of various bioactive components. The diverse chemical composition contained in this plant contributes to various biological activities, which may be beneficial for the health, food, and cosmetic industries. The purpose of this review was to summarise updated research on the in vitro and in vivo activities of *C*. *caesia* as well as the current clinical investigations. A compilation of the latest findings regarding the potential activities of *C*. *caesia* and mechanisms related to its health benefits is discussed and reviewed. This valuable information is the key that can be used for the development of drugs, functional food ingredients, and food products.

## 1. Introduction

Plants are the main source of natural products, which have been used for more than thousands of years for various purposes, including foods, traditional remedies, and ritual activities. Specialized metabolites produced by plants as natural defence mechanisms against diseases, infections, and predators have shown potential to be exploited for the development of new drugs. According to the World Health Organization (WHO), over 80% of the world's population uses herbal and natural products as medicinal treatment [1]. Besides, plenty of research findings revealed the beneficial therapeutic effects of various herbal plants and their extracts [2]. The use of herbal plants has gained interest from many sectors and in combating diseases because of their safety, availability, and low cost [3, 4].

The use of modern synthetic drugs has detrimental effects on the liver and/or kidney for long-term use, besides some having uncomfortable side effects. Turmeric is one of the ancient herbs that serves as a spice and possesses pharmacologic effects that have been proven in in vitro and in vivo studies. Urbanisation changed the lifestyle of people, which leads to increasing cases of numerous diseases. Various studies have been performed on the therapeutic properties of *Curcuma* species, and the ones mentioned here are merely a representation of them. *Curcuma caesia* contains beneficial components that can be exploited as natural complementary and alternative therapies to drugs for combating diseases, especially in humans.

**Figure 1:**
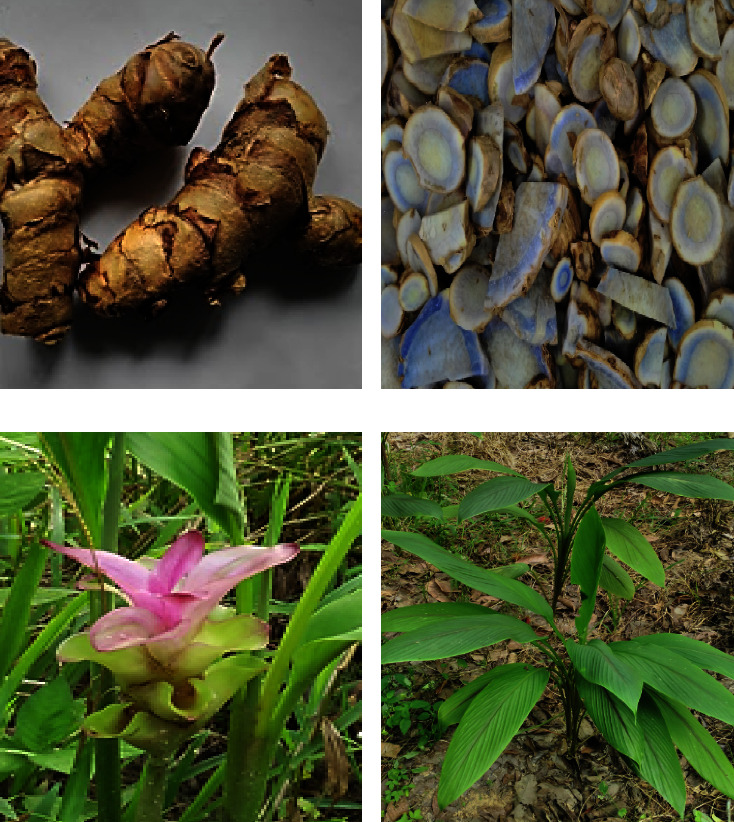
Rhizome (a), transverse cut of rhizomes (b), flower (c), and the whole plant of *C*. *caesia.* Roxb. (d).

Table 1 shows the classification of *C*. *caesia* according to the data from the Global Biodiversity Information Facility [5]. *Curcuma* (*Zingiberaceae*) consists of more than 80 species of rhizomatous herbs that are mostly native to tropical South Asia. Among the *Curcuma* genus, *C*. *caesia* is still considered an underutilised species, which has not been widely studied or commercially exploited yet. *C*. *caesia* is a perennial rhizomatous plant that erectly grows to a height of 0.5–1.0 m. It has a large tuberous rhizome, broad, vertical oblong leaves, and a pale yellow with reddish border flower [6]. The inner part of the rhizome is bluish-black or buff in colour and has a circular arrangement that is frequently misunderstood as the growth ring [7].  Figure 1 shows the rhizome (a), transverse cut of rhizomes (b), flower (c), and whole plant of *C*. *caesia* (d).

*C. caesia*, known as kali haldi (Hindi), black turmeric (English), and kunyit hitam (Malaysia), grows in humid, rich, and clayey soils. It originated throughout the Himalayan region, such as India and South East Asia. Even though there is no reliable report on the use of this plant as food material, its rhizome has been vastly reported to be used as a remedy in treating wounds, leprosy, bruises, toothache, snake and scorpion bites, ulcer, leucoderma, asthma, piles, bronchitis, and others [7]. The leaves were also found to be beneficial as rice seed germination stimulants, while the dried leaves can act as a fuel source [8]. This plant is currently considered an endangered species, which prompts the initiative of researchers to invent new approaches, such as plant cells and tissue culture in order to conserve the species from extinction [9]. This plant is considered an endangered species due to its traditional human usage, low multiplication rate in the soil, and root susceptibility to disease caused by the parasitic fungi, Pythium species [10]. Thus, this review was intended to summarise the most recent research on the in vitro and in vivo activities of *C*. *caesia* as well as the most recent clinical studies.

## 2. Traditional Uses

**Figure 2:**
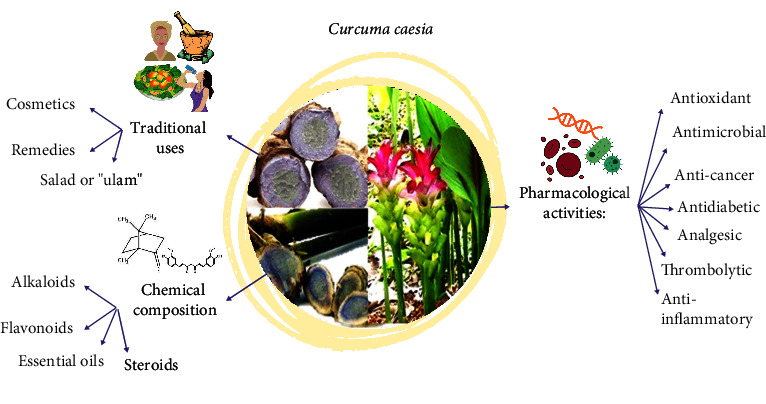
Graphical representation of traditional uses, chemical composition, and pharmacological activities of the *C*. *caesia* Roxb.

*C. caesia* has been used for a long time as a traditional herb in the Ayurvedic, Unani, and Siddha herbal systems [11].  Figure 2 summarises the overall information about *C*. *caesia*. Each part of the plant, especially the rhizomes and leaves, contained a different distribution of chemical constituents, which are used medicinally to combat various illnesses. Besides, *C*. *caesia* is also used in folk cosmetics and traditional medicine. Various parts of this plant are used for different purposes. In Kota Belud, Sabah (Malaysia), young leaves of *C*. *caesia* are often consumed raw as salad (ulam) by the Sama-Bajau people. The leaves are freshly eaten or given as a drink to treat cough [12].

The usage of this plant is more focused on the rhizome part. The presence of chemical compounds makes it aromatic and able to act as a stimulant and carminative agent. The rhizomes are employed as rubefacients by the “Turkomans” to rub their bodies after Turkish baths. In its fresh state, it is used as a turmeric replacement in Bengal, India [13]. Fresh rhizome is crunched and used as paste for sprains and bruises as well as migraine reliever [14]. Rhizome paste is also used to treat snake or scorpion bites. Powdered *C*. *caesia* rhizome is applied by tribal women as a facial mask during their engagement and marriage ceremonies [6]. In addition, it is mixed with honey or milk to be taken twice daily for longevity, weakness, infertility, and irregular menstrual flow [15]. It is also orally administered with water for bloating and stomach ache [14], while an extract prepared from dried rhizomes is taken orally for asthma relief [13].

Other uses of this plant are reported for the treatment of bronchitis, cold, cancer, epilepsy, fever, wounds, leucoderma, pneumonia, piles, tumor, toothache, vomiting, and gout [6, 14–16]. Nevertheless, despite its uses in humans, *C*. *caesia* is also used in animal healthcare, where rhizome juice is mixed with mustard oil and given to cattle with dysentery once daily on an empty stomach for 2-3 days [16]. Various studies have been conducted on this plant to investigate the scientific basis for its traditional usage, especially in disease therapies.  Figure 2 and Table 2 display the previous findings of the biological activities by *C*. *caesia*.

## 3. Phytochemical and Bioactive Constituents

The colour, odour, and taste of plants are contributed by the specialized metabolites that serve as the plant defence mechanism against adverse conditions caused by pathogens, herbivory, and environmental stress [43]. The presence of various phytochemicals in plants contributes to their pharmacological activities. The biological effects of *Curcuma* species are mainly attributed to the compound known as curcuminoids. There are many published articles reporting the presence of specialized metabolites, such as curcuminoids in turmeric, which are mainly accumulated on the rhizomes. Curcuminoids are commonly used as spices, pigments (dye substances), and additives, as well as therapeutic agents [44]. It is a type of fat soluble composed of polyphenolic pigments, the main component of which is curcumin. Beside curcumin, there are other components in the curcuminoids group, which are desmethoxycurcumin, bisdemetoxycurcumin, and cyclic curcumin. Among these, curcumin is the major component, and cyclic curcumin is the minor component [45]. Curcuminoids are also known as diferuloylmethane, which can be found mainly in the rhizome of *C. longa* and in other *Curcuma* species. It is insoluble in water and soluble in acetic acid, alkalis, chloroform, ethanol, and ketone [44, 46].

In a previous study, the presence of curcumin in the ethanolic extract of *C*. *caesia* (8 mg/100 g) by Soxhlet extraction method was the lowest among the other five *Curcuma* species: *C*. *longa* (125 mg/100 g), *C*. *zedoaria* (88 mg/100 g), *C*. *angustifolia* (71 mg/100 g), *C*. *leucorrhiza* (5 mg/100 g), and *C*. *amada* (11 mg/100 g) [47]. An earlier comparative study [47] on thin layer chromatographic (TLC) profiling of two *Curcuma* species, *C*. *longa* and *C*. *caesia*, showed the presence of curcuminoids in the rhizomes of both *Curcuma* species. However, only leaves methanolic extract of *C*. *longa* showed distinct bands for curcuminoids. In *C*. *caesia*, only curcumin was detected, whereas desmethoxycurcumin and bisdemetoxycurcumin were absent. Abundance of research have been conducted and showed the biological effects of curcuminoids, including antioxidant [48], antitumor [49], anti-inflammatory [50], and neuroprotective effects [51].

*C. caesia* was reported to be rich with major constituents of carbohydrates, proteins, amino acids, steroids, glycosides, flavonoids, alkaloids, tannins, and phenols [6, 11, 52–55]. Furthermore, this plant contains essential oils with camphor, ar-turmerone, (Z)-*β*-ocimene, and ar-curcumene as major compounds (higher than 5%), which produce the aromatic, hot, bitter taste, and pungent smell [56]. These metabolites are believed to contribute to the various biological activities of *C*. *caesia*. The differences in type and quantity of extracted compounds may be due to factors in the plant sample or extraction techniques, including plant sample preparation, type and polarity of solvent, temperature, pH, particle size, pressure, and time, as well as the origin of the plant [57–59]. The phytochemical constituents of *C*. *caesia* extracted from rhizomes and leaves using different types of extraction methodologies are summarized in the supporting information (Table S1 and Figure S1).

## 4. Antioxidant Properties

The therapeutic effects of some plant parts, such as fruits, vegetables, and grains, which are traditionally used in folk medicine, are usually attributed to their antioxidant compounds [60]. The protective role of plant phytochemicals may be associated with their antioxidant activity, as they combat oxidative stress in the body by maintaining a balance between oxidants (reactive oxygen species and reactive nitrogen species) and antioxidants [61]. Hence, the antioxidant compounds from natural products constitute the major source of human health promotion and maintenance [62]. In vitro and in vivo studies conducted on *C*. *caesia* extracts discovered their antioxidant activity.

There were various antioxidant assays tested on *C*. *caesia*, such as 2,2-diphenyl-1-picrylhydrazyl (DPPH), ferric reducing activity (FRAP), total antioxidant capacity, thiobarbituric acid reactive species (TBARS) assay, and total phenolic content (TPC). These different assays measure different antioxidant mechanisms; for example, the DPPH assay detects the scavenging activity by donating an electron (primary antioxidant), whereas the FRAP assay determines the ability to reduce ferric to ferrous by donating an electron (primary and secondary antioxidant) [63, 64]. Secondary metabolite content in plants is claimed to be the reason for the effectiveness of the plants as herbal medicine. According to Zayapor et al. [65], dietary antioxidants are majorly attributed to phenolic compounds. The amount of polyphenol and antioxidant ability of each *Curcuma* species varies; however, previous reports revealed yellow turmeric (*C*. *longa*) has higher phenolic content and exhibits stronger antioxidant activity, followed by white (*C*. *zedoaria*) and black turmeric (*C*. *caesia*) [66, 67]. Conversely, protein extracts from *C*. *caesia* rhizomes were reported to exhibit higher antioxidant activity by showing the lowest inhibition concentration (IC_50_ value) in the DPPH assay compared to *C*. *zedoaria* [68].

A study by Liu et al. [27] reported that methanolic *C*. *caesia* rhizome extract was a good source of natural antioxidant. It was discovered that methanolic extract of *C. caesia* rhizome at 100 *μ*g/mL profiles exhibited similar oxidative effects when compared to vitamin C and tert-butylhydroquinone (TBHQ) at 25 *μ*g/mL in [3-(4,5-dimethylthiazole-2-yl)2,5-diphenyltetrazolium bromide] (MTT assay) and moderately inhibited lipid peroxidation activity up to 43% [27]. In addition, a previous study showed significant effectiveness of a methanolic extract of *C*. *caesia* rhizome in scavenging reactive oxygen species (ROS) and reactive nitrogen species (RNS) in a dose-dependent manner [21]. The phenolic content in 10 mg of the extract was reported with value of 677.7 *μ*g, which accounted for the antioxidant activity against free radicals with IC_50_ values of 21.07 ± 1.78, 33.33 ± 0.52, 68.10 ± 1.24, 94.03 ± 0.67, 155.59 ± 3.03, and 260.56 ± 12.65 *μ*g/mL for hydroxyl, hypochlorous acid, superoxide, DPPH, nitric oxide, and peroxynitrite, respectively. Moreover, a study found that 50% methanolic extract of *C*. *caesia* leaves exhibited antioxidant activity through the TBARS inhibition assays with the range of 47.24–73.30% inhibition [69].

The type of solvent used for plant extraction plays an important role by affecting the extraction efficiency of the target compounds and influencing the activities of the extracts [69–71]. A study demonstrated that different polarities of solvents (hexane, petroleum ether, benzene, chloroform, ethyl acetate, methanol, and water) had significant effects on the total phenolic contents, extracted components, and antioxidant activities of fresh and dried *C*. *caesia* rhizomes [41]. The study reported chloroform and benzene extracts contained the highest TPC, with values ranging from 56.64–109.41 mg GAE/g extract. In addition, chloroform extract was revealed to be the most effective extract among the rest as free radical scavengers (DPPH assay), electron-donating agents (ferric reducing activity), and reducing molybdate ions (total antioxidant capacity). Starch isolated from *C. caesia* rhizomes through a cellulase-assisted method was reported to contain 4.1 mg ferulic acid equivalent (FAE)/g starch of total phenolic compounds, with only 0.015 mg/g starch curcumin content [30]. Besides, curcuminoid pigment (oleoresin) from *C*. *caesia* starch also showed antioxidant property by successfully scavenging 14.7% of free radicals in the DPPH assay [30].

The antioxidant property of this herb was also detected through in vivo studies. A report by Majumder et al. [17] revealed the antioxidant effectiveness of a methanolic extract of *C*. *caesia* rhizome by successfully scavenging free radicals in streptozotocin (STZ)-induced diabetes in Wistar rats. Lipid peroxidation was inhibited in diabetic rats treated with methanolic *C*. *caesia* extract by the reduction of TBARS level towards normal. Inhibition of lipid peroxidation helps in preventing tissue injury and failure of the endogenous antioxidant defence mechanisms. Besides, gradual healing was seen in the beta cells (pancreas) of the treated rats after treatment with methanolic *C*. *caesia* extracts.

## 5. Antimicrobial Properties

### 5.1. Antibacterial Properties

In some countries, bacterial infection is an epidemic. Exploring the antibacterial effect of *Curcuma* species has been conducted for many years by researchers, including on Gram-positive and Gram-negative bacteria. The most common bacteria used in the studies were *Bacillus subtilis*, *Staphylococcus aureus*, and *Escherichia coli*. Among them, *E. coli* was the most resistant strain [72, 73].

*C. caesia* leaves extracted with 50% methanol showed a positive antibacterial effect after 4 to 8 hr of incubation with *Bacillus cereus*, *Diplococcus pneumoniae*, *Streptococcus pyrogens*, and *Micrococcus glutamicum* [29]. *C*. *caesia* was revealed to have antibacterial properties by acetone extract, which showed maximum activity against *S*. *aureus*, while chloroform extract showed maximum inhibitory action against *Serratia marcescens* [31]. Research by Pandey and Gupta [40] reported that the rhizome extracts (chloroform, acetone, and methanol) of *C*. *caesia* were found to be more effective in inhibiting the bacterial growth as compared to stem and leaf extracts, excluding aqueous extract. The rhizome extracts of the plant showed inhibition against *Bacillus cereus*, *B. subtilis*, *S. aureus*, *Staphylococcus epidermidis*, *E. coli*, *Proteus vulgaris*, and *Klebsiella pneumoniae*. Meanwhile, in other findings by Borah et al. [42], essential oils from *C*. *caesia* leaves displayed an antibacterial effect against *B*. *subtilis*, *B*. *cereus*, *S*. *aureus*, and *Salmonella typhimurium*, with the lowest minimum inhibitory concentration (MIC) observed against *S*. *aureus* by 2.5 *μ*L.

*C. caesia* was found to exhibit significant in vitro antituberculosis activity [32]. An ethanolic extract of *C*. *caesia* rhizome was revealed have an effective antimycobacterial activity against *Mycobacterium tuberculosis* (Mtb H37Rv) and six multidrug-resistant (MDR) clinical strains of Mtb isolated from sputum samples of pulmonary tuberculosis (TB) patients with MIC of 31.25–125 *μ*g/mL. The same author also reported in vitro cytotoxicity of the extract in human THP-1 macrophages with IC_50_ value of 500 *μ*g/mL and 4–16 selective index range.

### 5.2. Antifungal Properties

Aflatoxin is a toxin produced by certain kinds of fungi (moulds) that are found naturally all over the world; they can contaminate food crops and cause serious health threats to humans and livestock [74]. *Curcuma* extract has been reported to be rich in bioactive compounds, which can act as antifungal agents [11, 14, 75]. The rhizome part of *C*. *caesia* was reported to have antifungal activity. Essential oil from *C*. *caesia* rhizome was tested against a number of human and plant pathogenic fungal strain. An ethanolic extract of *C*. *caesia* rhizome was detected to be effective in inhibiting the growth of *Aspergillus flavus* [34]. Other study discovered the antifungal activity by the isolated terpenoid (Z)-7-methoxy-1, 5-dihydrobenzo[c] oxepine from ethanolic *C*. *caesia* extract against *Fusarium oxysporum*, *Botrytis cinerea*, and *Rhizopus oryzae* [35]. Diethyl ether fraction of an extract mixture of *C*. *caesia* and *Ixora coccinea* showed better antifungal potential against *Botrytis cinerea* compared to any extract alone [33]. Nevertheless, a study by Borah et al. [42] on the *C*. *caesia* leaf essential oil also found it to have an antifungal effect against *Aspergillus fumigatus*, *Aspergillus niger*, *Saccharomyces cerevisiae*, and *Candida albicans*.

## 6. Antiproliferative and Anticancer Properties

Cancer is one of the major causes of death worldwide and may be developed due to several factors such as an unhealthy diet, genetic predisposition, or environmental factor. Most of the cases are caused by individual lifestyles and take about 20–30 years to develop [76]. The variety of biologically active components in natural substances, especially plants, were proven to have anticancer properties. The mechanism of action is through suppressing the inflammatory processes that lead to transformation, hyperproliferation, and the initiation of carcinogenesis [77]. Their inhibitory effects may eventually suppress the final steps of carcinogenesis: angiogenesis and metastasis [78].

Methanolic extract of *C*. *caesia* rhizomes showed positive effect of anticancer against Ehrlich's ascites carcinoma (EAC) in mice by significantly reducing the tumor volume, tumor weight, viable cell count and increasing the lifespan percentage (57.14 and 88.09%) of EAC-treated mice [36]. The antitumor activity may be influenced by the cytotoxic effect and antioxidant property of the extract tested. It was assumed that the viability of EAC cells decreased as the oxidative stress in different tissues in EAC-bearing mice was reduced. Research on the anticancer effect of *C*. *caesia* rhizomes exposed the effectiveness of *C*. *caesia* extract against the hepatocarcinogen, diethylnitrosamine (DEN), by enhancing antioxidant status through free radical scavenging mechanisms resulting in attenuation of hepatic enzymes in the serum (aspartate aminotransferase (AST), alanine aminotransferase (ALT), alkaline phosphatase (ALP), and cancer marker enzyme acetylcholine esterase (AChE)) [37]. The activities of the antioxidant defence system such as superoxide dismutase (SOD), catalase (CAT), glutathione peroxidase (GPx), and glutathione (GSH) were significantly increased in extract-treated mice when compared to controls.

Besides having anticancer property, *C*. *caesia* also has the potential to be used as a supplement for cancer treatment with a chemotherapeutic drug (cyclophosphamide) due to its capability to prevent drug toxicity through the alleviation of micronuclei formation and to prevent hepatotoxicity and nephrotoxicity caused by the drug [38]. The latest finding [39] revealed that hexane rhizome extract of *C*. *caesia* exhibited remarkable antioxidant activity with 1200 mg ascorbic acid equivalent/100 g. The extract was also able to inhibit HepG2 (human liver carcinoma cells) cell proliferation with very low IC_50_ value (IC_50_: 0.97 *μ*g/mL), high selective index, and induce cell arrest at the S and G2/M cell cycle phases along with caspase-3-mediated apoptosis.

## 7. Anti-Inflammatory and Antiulcer Properties

**Figure 3:**
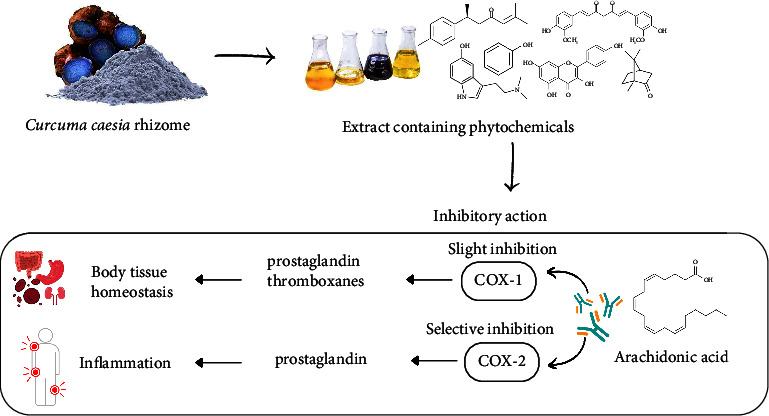
Mechanism of action of anti-inflammatory activity of *C*. *caesia* rhizome extract.

Inflammation is a biological response of the immune system that can be triggered by various factors, which may induce acute and/or chronic inflammatory responses in the body systems and organs, potentially leading to tissue damage or disease [79]. One important key role in the process of inflammatory is played by prostaglandins [27, 80]. Inflammatory disease can be reduced or delayed by inhibition of cyclooxygenase (COX) enzymes associated with inflammatory intermediates such as prostaglandin and thromboxanes [27]. Anti-inflammatory activity of hexane, methanolic, and pure isolate compounds from extract of *C*. *caesia* has been positively shown by selective inhibition of COX-2 and slight inhibition activity against COX-1 [68] and is summarized in supporting information (Figure 3). An in vivo study of methanolic *C*. *caesia* extract also showed its effectiveness as an anti-inflammatory agent, whereby at doses of 200 and 400 mg/kg, it significantly reduced the paw edema volume in carrageenan-induced paw edema in rats. The extract also demonstrated anti-inflammatory activity in a cotton pellet-induced granuloma model (subacute) by reducing the dry weight of granuloma [19]. Besides that, anti-inflammatory action also can be examined through the egg albumin denaturation assay. A study conducted by Borah et al. [42] discovered that at a concentration of 300 *μ*g/mL, *C*. *caesia* leaf essential oil exhibited the highest albumin denaturation inhibition activity and had lower IC_50_ value (182.5 *μ*g/mL) compared to the standard sodium diclofenac (906.5 *μ*g/mL).

Meanwhile, ulcer is a condition of an inflammation breakout in the skin or the alimentary tract mucus membrane lining. Production of free radicals, decline of mucosal defensive factor, or internal injuries are among the factors that contribute for the ulceration to take place [81, 82]. Various other herbal plants have been reported to possess antiulcer activity, including *Euphorbia umbellate* (leitosinha) stem bark [83], *Osyris quadripartita* leaves [84], *Filipendula ulmaria* (L.) Maxim, and *Filipendula vulgaris Moench* flower [85]. Previous literature claim that the bioactive components in herbal plants such as flavonoids, saponin, tannins gums, and mucilages are possible gastroprotective agents [86].

Efficiency of flavonoids and tannins as antiulceronic substances among the cytoprotective active materials has been extensively confirmed, whereas other components with other possible mechanisms such as alkaloid can inhibit stress-induced ulcer, and tannins may inhibit ulcer development due to their protein precipitating and vasoconstriction effects, which the astringent action can help precipitating microproteins on the ulcer site, thereby forming an impervious layer over the lining that hinders gut secretions and protecting the underlying mucosa from toxins and other irritants [87]. According to Mehta [88], the most common types of ulcer are duodenal, peptic, and gastric ulcer. *C*. *caesia* has significant ulceroprotective effect against gastric ulcer, which is comparable to standard drug ranitidine [28]. A significant reduction of ulcer index, gastric acid volume, pepsin, free and total acidity, along with increased production gastric mucus was detected in rats treated with ethanolic extract of *C*. *caesia* (EECC-500 mg/kg). The lethal dose value, LD_50_, for the extract was found to be higher than 2000 mg/kg.

## 8. Antidiabetic Properties

Sedentary lifestyle, rapid urbanization, and nutrition transition lead to the raising of global diabetic and obesity cases [89–91]. Improper control may cause damage to the heart, blood vessels, eyes, kidneys, and nerves, which leads to disability and premature death [92]. These complications are correlated with postprandial hyperglycemia, which are known due to the induction of oxidative stress causing a decline in enzyme antioxidant activity and the excessive production of free radicals [93–95].

According to Majumder et al. [17], the rhizome of *C*. *caesia* was found to be a potential antidiabetic agent when studied in STZ-induced diabetic rats, where it significantly reduced fasting blood glucose (FBG), glycosylated haemoglobin (HbA1c), and oral glucose tolerance test (OGTT) level towards normal as well as improved body weight when compared to the diabetic control group. They also reported methanolic *C*. *caesia* extract successfully inhibiting *α*-amylase and *α*-glucosidase with IC_50_ values of 442.92 ± 10.05 *μ*g/mL (acarbose = 154.33 ± 9.08 *μ*g/mL) and 95.40 ± 9.74 *μ*g/mL (acarbose = 38.63 ± 8.05 *μ*g/mL), respectively. The extract also showed potent glucose uptake activity in yeast cells by promoting the facilitated diffusion process. The mechanism involved in effectiveness activity of methanolic *C*. *caesia* rhizome extract in the experimental rat model might be by augmenting the endogenous antioxidant mechanism [17]. Recently, Jain and Parihar [18] investigated the antidiabetic activity of successive extracts of *C*. *caesia* rhizome. The results indicated that ethyl acetate extract showed relatively much higher inhibition activity in *α*-amylase inhibition assay (97.72% ± 0.28 and IC_50_ value: 63.96 *μ*g/mL) compared to curcumin and camphor as standards. They also discovered the chloroform : methanol (80 : 20) fraction of *C*. *caesia* ethyl acetate to contain eucalyptol, camphor, and germacrone as antioxidant property as well as caryophyllene and *β*-sitosterol with antidiabetic and antioxidant properties.

## 9. Neuropharmacological Properties

### 9.1. Analgesic Effect

Analgesic activity works as a painkiller or pain reliever, which is mainly used in treating or controlling numerous diseases. Analgesic agents act on the peripheral and central nervous systems by several mechanisms of action without causing loss of consciousness [96]. Methanolic extract of *C*. *caesia* is suggested to be a peripherally and centrally acting analgesic in Sawant et al.'s [19] study, in which the extract showed significant (*p* < 0.001) activity in the acetic acid-induced writhing mice model as well as in the hot plate test at doses of 200 and 400 mg/kg. Mice treated with extract at 100, 200, and 400 mg/kg significantly (*p* < 0.001) decreased the number of writhing by 11.94%, 22.38%, and 31.34%, respectively, when compared to the control group. Their observation found that the extract dose-dependently increased the pain latency time in response to the thermal stimulation procedure. The presence of phytochemical compounds in the extract such as alkaloids, flavonoids, phenols, saponins, and tannins, may contribute to the observed activity [19]. A previous report by Karmakar et al. [22] also found analgesic activity in *C*. *caesia* by acetic acid-induced writhing and tail flick tests, which demonstrated significant peripheral antinociceptive actions.

Other findings revealed that treatment with curcumin was able to lessen the thermal hyperalgesia and mechanical allodynia, as measured by an extended latency to paw withdrawal (PWL) from a noxious thermal stimulus or a mechanical stimulus. A study by Cheppudira et al. [97] reported that curcumin blocked heat-induced secretion of GRO-a and IL-8, both at basal levels, as well as heat-induced matrix metalloproteinases 1 and 3 (MMP-1 and MMP-3). Furthermore, curcumin was found to suppress p38 MAPK activity and heat-inducedNF-kB p65 activity, which indicates that curcumin may be an effective analgesic for thermal injury pain through suppression of inflammation at the site of injury.

### 9.2. Anticonvulsant, Muscle Relaxant, and Locomotor Depressant

Previous findings by Arulmozhi et al. [23] found the muscle relaxant property of *C*. *caesia* hydroalcoholic extract in the bronchioles and vasculature of the respiratory tract in rodent models. The mechanism of action was suggested to be the ability of extract to inhibit calcium release from intracellular calcium stores and also the calcium efflux from extracellular space. Other finding reported locomotor depressant, anticonvulsant, and muscle relaxant activity of *C*. *caesia* rhizome [22]. Locomotor activity in mice was significantly (*p* < 0.001) depressed in a dose-dependent fashion by a methanolic extract of *C*. *caesia*, which indicates that the extract has CNS property in mice. The extract also was found to significantly protect mice from pentylenetetrazole (PTZ)-induced convulsions by delaying the onset of convulsions and allowing the animals to recover, leading to survival. Meanwhile, in a muscle relaxant study, the extract significantly (*p* < 0.001) and dose-dependently reduced the fall-off time in mice, reflecting its muscle relaxant property. In the muscle relaxant assay, the methanolic *C*. *caesia* extract induced a decrease in fall-off time due to the loss of muscle grip, implying muscle relaxation. The results show extract induced neurological deficit by its taming or calming effect in mice, thus supporting its role of CNS-depressant [22].

### 9.3. Anxiolytic and CNS Depressant

The research conducted by Karmakar et al. [20] examined methanolic extract of *C*. *caesia* rhizome capacity for hypnotic activity, forced swim test (FST), and tail suspension test (TST) performed in Swiss albino mice models. Their study revealed that the extract significantly induced longer sleeping times and showed antidepressant activity in TST and FST. They also suggested that possible mechanism of action was through the elevation of monoaminergic neurotransmitters levels in the brain due to the presence of monoamine oxidase nonselective inhibitor and attenuation of oxidative stress produced during depression, by the tannic acid present in the extract [20].

## 10. Thrombolytic Properties

A blood clot causes blockage or occlusion of a blood vessel that leads to various life-threatening diseases such as atherosclerosis, glaucoma, heart attack, and stroke. Thrombolytic agents assist in dissolving the blood clot and reduce the risk of the mentioned diseases [98, 99]. A study by Fathima et al. [24] found that the ethanolic extract of *C*. *caesia* showed effective properties in thrombolytic activity, whereby it significantly exhibited cell lysis by 49.18 ± 3.41% as compared to the positive control, streptokinase (71.54 ± 3.26%), and the negative control, water (2.96 ± 0.28%). Meanwhile, Bharathi et al. [25] discovered that the hydroalcoholic extract of *C*. *caesia* has the ability to exhibit thrombolytic activity by 38.75%, which was slightly higher compared to *C*. *amanda* with 34.74%. Moreover, the silver nanoparticle of *C*. *caesia* also showed high activity in lysing the blood clot.

## 11. Toxicity Study of *C*. *caesia*

The toxicity of substances can be observed via study of the accidental exposures to a substance, in vitro studies using cells/cell lines, and in vivo exposure on animal models [100]. Previous toxicity studies of *C*. *caesia* were mainly conducted in vitro and in vivo. As mentioned above, in-vitro toxicity studies of the *C*. *caesia* extract have been done against EAC [101], human laryngeal cancer (Hep 2), human liver adenocarcinoma (HepG2), human colon adenocarcinoma (HT 29), monkey kidney (VERO) [102], and human acute monocytic leukemia (THP-1) cell lines [32], which all demonstrated the toxicity effects of the plant. Meanwhile, acute toxicity studies in experimental animals (mice and rats) have been done up to several high doses, including 1000 [37], 2000 [17, 19, 28], and 3000 [36] mg/kg body weight per oral administration. All reported survival of the animals after administration of the plant extract, thus revealed that the LD_50_ is higher than that amount of dose tested.

Besides, the toxicity of a substance can also be monitored through biochemical parameters, such as the liver and kidney marker enzymes that are commonly used to quantitatively evaluate the degree of damage to the organs [76]. A study by Hadem et al. [37] reported that diethylnitrosamine (DEN)-treated mice showed a significant reduction of serum transferases (alanine aminotransferase (ALT), aspartate aminotransferase (AST), and alkaline phosphatase (ALP)) towards their control as observed after 16 and 28 weeks. This result tallied with a study by Devi and Mazumder [38] that reported significant reductions in SGOT and SGPT levels in cyclophosphamide (CP)-treated mice compared to the positive group and another study by Majumder et al. [17] that reported that the ALP level in the *C. caesia*extract-treated group was significantly (*p* < 0.05) lowered compared to the STZ control group.

Another form of toxicity study, known as the *Allium cepa* assay, is chemical screening and in situ monitoring for genotoxicity of environmental contaminants, such as pesticides, which help detect if these compounds can induce chromosomal aberrations in the root meristems of *A. cepa* [103, 104]. Borah et al. [42] discovered that *C*. *caesia* leaf essential oil possesses a negligible amount of genotoxic action towards root growth test, mitotic index, and chromosomal aberrations in the *Allium cepa* assay, as compared to the positive control ethyl methanesulfonate (EMS). They also claimed that the *C*. *caesia* leaf essential oil is potentially safe to be used in pharmaceutical and industrial applications.

## 12. Effort on Plant Conservation

This plant is currently considered as endangered species due to the extensive traditional human usage, low multiplication rate in the soil, root susceptibility to disease caused by parasitic fungi, Pythium species [10], as well as industrialization and urbanisation, which destroyed their natural habitat [105]. However, a new propagation method is needed for this plant due to the minus points of the conventional method, including (i) that 10–20% of the yield is required for raising the next crop, (ii) the risk of soil-borne diseases from one crop to the next and from one location to another, and (iii) the necessity of proper preservation of the seed rhizomes during the gap period between harvesting and raising the next crop, as they are prone to rhizome rot caused by bacteria, fungi, and insect attacks [106]. Currently, in vitro cell and tissue culture methodologies are widely used as a means for plant conservation to ensure the survival of endangered species. Besides, this method requires only small explants for micropropagation and high multiplication rates for large-scale revegetation and is able to produce disease-free plants [107]. Thus, in order to conserve the species from extinction, initiatives have been taken by researchers through plant cells and tissue culture approaches [9].

## 13. Conclusion

Most people are now shifting from using synthetic to natural-based products due to the safety issue. Medicinal plants and herbs are studied extensively, leading to numerous promising discoveries. *C*. *caesia* is one of the herbal plants currently gaining popularity among researchers due to its potential health benefits. Overall, it can be concluded that *C*. *caesia* has the potential to be explored as a natural complementary and alternative health therapy with potential for the pharmaceutical and nutraceutical industries. Research reports on *C*. *caesia* suggest its medicinal and pharmaceutical potentials might be due to the variety of metabolites in the plant that give the effects observed, which can be determined by an advanced metabolomics approach. Therefore, authors are currently investigating the plant metabolites through the NMR analysis and bioactivity variations associated with different solvent ratios that hypothesized absolute ethanol extract to be most excellent extract with abundance of metabolites, which are responsible for the bioactivities [108, 109].

The uses of *C*. *caesia* due to its potential health benefits to humans are gaining interest by researchers. Several scientific studies have found the antioxidant, antibacterial, antifungal, antiproliferative, anticancer, anti-inflammatory, analgesic, and anticonvulsant properties as well as the muscle relaxant, locomotor depressant, antidepressant, and thrombolytic activities of *C*. *caesia*. Further studies are required to identify, isolate, and elucidate the structures of novel bioactive constituents present in *C*. *caesia* that contribute to its health benefits. Besides, more evidence, especially through in vivo and clinical trials, is necessary to determine the mechanisms involved in the bioactivities mentioned previously. Even though there are available preliminary data on the nutritional values of *C*. *caesia* rhizomes, information is still lacking, especially on other parts of the plant, such as the leaves, stem, and flower. Thus, studies on the leaves, stem, and flower of *C*. *caesia* could also be done to discover potential bioactive compounds that can be exploited for health advantages.

## Figures and Tables

**Table 1:** Classification of *Curcuma caesia* [5].

Kingdom	Plantae, plants
Phylum	Tracheophyta, vascular plants
Class	Liliopsida, monocotyledons
Order	Zingiberales
Family	Zingiberaceae, ginger family
Genus	*Curcuma*
Species	*Curcuma caesia* Roxb.

**Table 2:** Medicinal and pharmacological properties of *Curcuma caesia.*

Part	Extraction/isolation methodology	Medicinal and pharmacological properties	Findings	References
Rhizome	Soxhlet extraction with methanol	Antioxidant, antidiabetic activity	IC_50_ values for *α*-amylase and *α*-glucosidase inhibition were found to be 442.92 ± 10.05 *μ*g/mL and 95.40 ± 9.74 *μ*g/mL, respectively. The extract also successfully scavenged superoxide and hydroxyl ions	Majumder et al. [17]
Rhizome	Soxhlet extraction with chloroform, dichloromethane, acetone, ethyl acetate, methanol, and ethanol	Antidiabetic activity	Chloroform and methanol fraction in the ratio of 80 : 20 showed highest *α*-amylase inhibition (IC_50_ = 80.13 *μ*g/mL)	Jain and Parihar, [18]
Rhizome	Soxhlet extraction with methanol	Analgesic, anti-inflammatory activity	Significant analgesic activity of *C. caesia* extract was observed in an acetic acid-induced writhing model and in a hot plate test. Extract also showed significant (*p* < 0.001) anti-inflammatory activity by reducing the paw edema volume in carrageenan-induced paw edema in rats in the late phase (3 to 5 h) and decreased the dry weight of granuloma	Sawant et al. [19]
Rhizome	Soxhlet extraction with methanol	Antioxidant, antidepressant activity	The IC_50_ values for scavenging free radicals for DPPH, nitric oxide, superoxide, hydroxyl, peroxynitrite, and hypochlorous acid were 94.03 ± 0.67 *μ*g/mL, 155.59 ± 3.03 *μ*g/mL, 68.10 ± 1.24 *μ*g/mL, 21.07 ± 1.78 *μ*g/mL, 260.56 ± 12.65 *μ*g/mL, and 33.33 ± 0.52 *μ*g/mL, respectively. In the in vivo test, *C. caesia* extract was observed significantly (*p* < 0.05) and reduced the onset and prolongation of rat sleep duration as well as decreased the immobility periods in both FST and TST	Kamarkar et al. [20]
				Kamarkar et al. [21]
Rhizome	Soxhlet extraction with methanol	Analgesic, locomotor depressant, anticonvulsant, and muscle relaxant activity	Extract significantly (*p* < 0.001) reduced the writhes with 75.71% and 90.39% protection compared to the control group. Treatment with the extract also exhibited 51.95% and 79.99% reduction in motor activity. For anticonvulsion activity, the onset of convulsions was significantly (*p* < 0.001) delayed, which led to the animal's survival. For muscle relaxant activity, the extract significantly (*p* < 0.001) and dose-dependently decreased the fall-off time in mice	Karmakar et al. [22]
Rhizome	Maceration extraction with ethanol	Smooth muscle relaxant activity	The extract concentration dependently relaxed the carbachol (1 *μ*M)-induced precontractions in isolated Guinea pig trachea with the IC_50_ value of 239.36 *μ*g/mL	Arulmozhi et al. [23]
Rhizome	Maceration extraction with ethanol	Thrombolytic activity	The ethanolic *C. caesia* extract was found to have significant thrombolytic activity (49.18 ± 3.41%) compared to the effect of streptokinase (71.54 ± 3.26%) used as the positive control and water (2.96 ± 0.28%) used as the negative control in the experiment	Fathima et al. [24]
Rhizome	Maceration extraction with alcohol and water	Thrombolytic activity	Percentage of clot lysis by *C. caesia* rhizome extracts (38.75 ± 2.217%) and synthesized silver nanoparticle (51%) was statistically significant (*p* < 0.05) compared to the positive control streptokinase and negative control water	Bharathi et al. [25]
Rhizome	Hydrodistillation	Antioxidant and antibacterial activities	*C. caesia* essential oil possessed 22.5 ± 0.12 *μ*g GAE/*μ*L oil content of phenolics, IC_50_ 6.3 ± 0.06 *μ*L DPPH scavenging activity, and EC_50_ 1.6 ± 0.1 *μ*L ferric reducing power. The essential oil also had antibacterial activity against *S. aureus*, *B. subtilis*, and *E. coli*	Angel et al. [26]
Rhizome	Soxhlet extraction with hexane and methanol	Antioxidant, anti-inflammatory, and tumor cell proliferation inhibitory activities	The hexane and methanolic extracts of *C. caesia* showed LPO inhibition by 31 and 43%, and COX-2 enzyme by 29 and 38%, respectively. The extracts also inhibited the growth of human tumor cells	Liu et al. [27]
Rhizome	Maceration extraction with ethanol	Antiulcer activity	*C. caesia* showed significant ulceroprotective effect against gastric ulcer in albino rats by reducing the ulcer index (4.18 ± 0.60), volume of gastric juice (1.14 ± 0.10 mL/4 hr), free (46.40 ± 2.13 mEq/L) and total acidity (66.80 ± 1.35 mEq/L), pepsin along with increased production of mucus	Das et al. [28]
Rhizome	Maceration extraction with hexane, petroleum ether, benzene, chloroform, ethyl acetate, methanol, and water	Antioxidant activity	The TPC value for the extracts were in the range of 26.43 ± 0.4–109.41 ± 0.36 mg GAE/g extract. Result also revealed that samples extracted with more polar solvents had higher antioxidant activity	Reenu et al. [29]
Rhizome	Isolation of starch with cellulase	Antioxidant activity	Total phenolic and curcumin contents and DPPH antioxidant capacity of *C. caesia* starches was 4.1 ± 0.2 mg FAE/g starch, 0.015 ± 0.001 mg/g starch, and 14.7 ± 0.3% scavenging, respectively	Hung and Duyen [30]
Rhizome	Soxhlet extraction with hexane, chloroform, ethyl acetate, acetone, methanol, and water	Antibacterial activity	Higher inhibitory action was detected against *S. aureus* (acetone extract, zone of inhibition = 22 ± 1.9 mm), and *S. marcescens* (chloroform extract, zone of inhibition = 27 ± 1.9 mm)	Thomas and Jose [31]
Rhizome	Maceration extraction with ethanol	Antimycobacterial activity	Ethanolic extract showed antimycobacterial activity with MIC value 125 *μ*g/mL against *M. tuberculosis* H37Rv, and cytotoxic effect on THP-1 macrophages with IC_50_ value 500 *μ*g/mL	Gupta et al. [32]; Ghosh [33]
Rhizome	Soxhlet extraction with ethanol, water	Antibacterial, antifungal activity	Ethanolic extract of *C. caesia* was effective against *A. flavus*, with 11 mm zone of inhibition recorded	Harit et al. [34]
Rhizome	Maceration extraction with ethanol	Antibacterial and antifungal activities	Antifungal assay showed the MIC values as 22 mg/mL (*Botrytis cinerea*), 27 mg/mL (*Fusarium oxysporum*), and 17 mg/mL (*Rhizopus oryzae*). The MIC values for the antibacterial effect was 267 mg/mL (*Serratia marcescens*), 291 mg/mL (*Erwinia herbicola*), 345 mg/mL (*Xanthomonas* sp.), and 467 mg/mL (*Arthrobacter chlorophenolicus*)	Ghosh et al. [35]
Rhizome	Soxhlet extraction with methanol	Antitumor and antioxidant activities	Methanolic extract exhibited cytotoxicity effect (IC_50_ 90.70 ± 8.37 mg/mL) on EAC cell line. Extract also significantly (*p* < 0.01) reduced tumor volume, tumor weight, viable cell count, and increased the lifespan percentage (57.14 and 88.09%) of EAC-treated mice	Karmakar et al. [36]
Rhizome	Maceration extraction with methanol	Anticancer activity	Treatment of mice with *C. caesia* extract attenuated the increased activities of the marker enzymes (AST, ALT, ALP, and AChE), which was caused by DEN administration	Hadem et al. [37]
Rhizome	Maceration extraction with methanol	Protective activity against genotoxicity	The methanolic extract was found to scavenge the free radicals ABTS^+^ (IC_50_ = 51.994 *μ*g/mL) and reduce the number of micronuclei (41.77–68.75%). The levels of serum SGPT, SGOT, GSH, and GR also decreased with the pretreatment of the extract	Mazumder and Devi [38]
Rhizome	Soxhlet extraction with hexane, ethyl acetate, methanol, and water	Antioxidant and anticancer activities	Hexane extract was found to possess remarkable antioxidant activity (1200 mgAAE/100 g) and dose-dependent inhibition in HepG2 cell lines (IC_50_ = 0.976 *μ*g/mL)	Mukuthan et al. [39]
Rhizome, leaves, stem	Soxhlet extraction with water, methanol, acetone, and chloroform	Antibacterial activity	The rhizome extracts were found to be more effective in inhibiting the bacterial growth as compared to stem and leaf. Methanol and chloroform extracts showed highest activity index against *B. cereus* (0.55) and *K. pneumoniae* (0.59), respectively	Pandey and Gupta [40]
Leaves	Maceration extraction with methanol	Antioxidant, antimicrobial, and immunomodulatory activities	Extract showed antibacterial effect against *B. cereus* (14.95 ± 0.71 mm), *D. pneumoniae* (14.65 ± 0.71 mm), *M. glutamicum* (12.50 ± 0.24 mm), and *S. pyogenes* (13.71 ± 0.41 mm). The extract also had the highest antioxidant activity (73.3 ± 0.45%) at the concentration of 250 *μ*g/mL and showed 15% macrophage yeast digestion	Bhardwaj et al. [41]
Leaves	Hydrodistillation	Antioxidants and anti-inflammatory and antimicrobial activity	Leaf oil contained phenolics (2.13 ± 0.027 mg/mL) and flavonoids (11.36 ± 0.096 mg/mL) and exhibited antioxidant (IC_50_ value = 1.487 *μ*g/mL), anti-inflammatory (182.5 *μ*g/mL), and antimicrobial potential against *B. subtilis*, *B. cereus*, *S. aureus*, *S. typhimurium*, *A. fumigatus*, *A. niger*, *S. cerevisiae*, and *C. albicans*	Borah et al. [42]

## Data Availability

The data used to support the findings of this study are included within the article. Any other data can be available upon request from the corresponding author.

## Abbreviations

AChE: Acetylcholine esterase
ALP: Alkaline phosphatase
ALT: Alanine aminotransferase
AST: Aspartate aminotransferase
CAT: Catalase
CNS: Central nervous system
COX: Cyclooxygenase
CP: Cyclophosphamide
DPPH: 2,2-Diphenyl-1-picrylhydrazyl
DEN: Diethylnitrosamine
EAC: Ehrlich's ascites carcinoma
EECC: Ethanolic extract of *C*. *caesia*
EMS: Ethyl methanesulfonate
FRAP: Ferric reducing activity
FAE: Ferulic acid equivalent
FBG: Fasting blood glucose
FST: Forced swim test
GAE: Gallic acid equivalence
GPx: Glutathione peroxidase
GSH: Glutathione
HbA1c: Glycosylated haemoglobin
IC_50_: Inhibition concentration
LD_50_: Lethal dose
MIC: Minimum inhibitory concentrations
MDR: Multidrug resistant
MTT: 3-(4,5-Dimethylthiazole-2-yl)2,5-diphenyltetrazolium bromide
OGTT: Oral glucose tolerant test
PWL: Paw withdrawal
PTZ: Pentylenetetrazole
STZ: Streptozotocin
SOD: Superoxide dismutase
SGOT: Serum glutamic oxaloacetic transaminase
SGPT: Serum glutamate pyruvate transaminase
TB: Pulmonary tuberculosis
TBARS: Thiobarbituric acid reactive species
TBHQ: Tert-butylhydroquinone
TPC: Total phenolic content
TST: Tail suspension test.

## Supplementary Materials

Supplementary Materials Supplementary Table S1. Phytochemical constituents of *C*. *caesia* extracted from rhizomes and leaves using different types of extraction methodologies. Supplementary Figure S1. Phytochemical constituents of *C*. *caesia.*
