# Supplementary material for: A Comprehensive Review with Future Prospects on the Medicinal Properties and Biological Activities of Curcuma caesia Roxb
Source: Evid Based Complement Alternat Med. 2023 Jan 17;2023:7006565. doi: 10.1155/2023/7006565 (PMC9873438; doi:10.1155/2023/7006565)
Supplement: Supplementary Materials — Supplementary Table S1. Phytochemical constituents of C. caesia extracted from rhizomes and leaves using different types of extraction methodologies. Supplementary Figure S1. Phytochemical constituents of C. caesia. [file 7006565.f1.docx]

**Table S1:** Phytochemical constituents of *C. caesia* extracted from rhizomes and leaves using different type of extraction methodology.

| **Part of plant** | **Extraction / isolation methodology** | **Phytochemical detected** | **Reference** |
| --- | --- | --- | --- |
| Rhizomes | Soxhlet extraction with methanol | Curcuminoids, flavonoid, phenol, alkaloid, buffer soluble protein, volatile oil | Sarangthem and Haokip [52] |
| Rhizomes | Soxhlet extraction with acetone and methanol | Curcumin | Verma et al. [15] |
| Rhizomes | Soxhlet extraction with methanol | Steroid, tannin, saponin, flavonoid, alkaloid | Karmakar et al. [20] |
| Rhizomes | Soxhlet extraction with n-hexane, petroleum ether (60:80), benzene, chloroform, ethyl acetate, methanol, and water | Carbohydrate, protein, fixed oils and fats, steroids and terpenoids, alkaloids, glycosides, tannins and phenols | Paliwal et al. [6] |
| Rhizomes | Soxhlet extraction with methanol | Camphor | Paliwal et. al. [6] |
| Rhizomes | Soxhlet extraction with ethanol | Nine sesquiterpenes with chemical skeleton types of carabrane, elemane, germacrane, and guainane. | Vairappan et al. [108] |
| Rhizomes | Soxhlet extraction with methanol and chloroform | Curcumin, dis-methoxycurcumin and bis-demethoxycurcumin | Behar et al. [54] |
| Leaves | Soxhlet extraction with methanol and chloroform | Curcumin | Behar et al. [54] |
| Rhizomes | Soxhlet extraction with methanol | Carbohydrate, steroids, flavonoids, alkaloids, phenols, tannins, glycoside, amino acid terpenoids and camphor | Lawand and Gandhi [109] |
| Rhizome | Isolation of starch with cellulase | Curcumin | Hung and Duyen [30] |
| Rhizomes | Soxhlet extraction with methanol | 2-Methylbenzene-1, 3-diol | Sahu and Saxena [14] |
| Rhizomes | Soxhlet extraction with methanol and chloroform | Carbohydrates, proteins, amino acids, steroids glycosides, flavonoids, alkaloids, tannins, phenols, and resins | Ranemma and Reddy  [11] |
| Rhizomes | Soxhlet extraction with methanol | α-santalol, retinal, ar-turmerone, alloaromadendrene, megastigma-3,7(e),9-triene, benzene, 1-(1,5-dimethyl-4-hexenyl)- 4-methyl, 5,8,11,14,17-eicosapentaenoic acid, methyl ester, (all-z)-, tricyclo [8.6.0.0(2,9)]hexadeca-3,15-diene, trans-2,9-anti-9,10- trans-1,10-, (+)-2-bornanone, isoborneol, trans-sesquisabinene hydrate, and androstenediol | Pakkirisamy et al. [110] |

**Table S1:** Continued.

| **Part of plant** | **Extraction / isolation methodology** | **Phytochemical detected** | **Reference** |
| --- | --- | --- | --- |
| Rhizomes | Soxhlet extraction with ethyl acetate | eucalyptol, camphor, caryophyllene, germacrone and β-sitosterol | Jain and Parihar [18] |
| Rhizomes | Soxhlet extraction with ethanol | alkaloid, flavonoid, tannin, terpenoid, saponin, phenolic, anthraquinone | Ramkumar et al. [111] |
| Rhizomes | Supercritical fluid extraction (SFE) | beta-elemene, curzerenone, boldenone, and 2-cyclohexen-1-one, 4- ethinyl -4-hydroxy-3, 5, 5-trymetyl | Chaturvedi et al. [112] |
| Rhizomes | Isolation of essential oil by hydrodistillation | camphene, p-cymene, 1,8-cineole, (z)-β-ocimene, 5-nonanone, α-terpinolene, linalool, endo-fenchol, camphor, menthone, isomenthone, borneol, terpinen-4-ol, estragole, endo-fenchyl acetate, bornyl acetate, δ-elemene, β-cubebene, β-elemene, (z,e)-α-farnesene, β-caryophyllene, γ-elemene, γ-curcumene, ar-curcumene, β-selinene, elemol, germacrene b, epiglobulol, viridiflorol, ar-turmerone | Pandey and Chowdhury  [56] |
| Rhizomes | Isolation of essential oil by hydrodistillation | Eucalyptol, camphor, isoborneol, borneol, α-terpineol, isobornyl acetate, β-elemene, caryophyllene, α-selinene, α-bulnesene, δ-cadinene, caryophyllene oxide, γ-cadinene, rosifoliol, tropolone, cis-α-copaene-8-ol, β-selinene, β-guaiene, globulol, ledol, ar-turmerone, spathulenol, 2,7-dimethyl oxepine, β-elemenone, occidentalol, cyclohexanol, 1,3,3-trimethyl-2- (3-methyl-2-methylene-3-butenylidene)-(2z)6-isopropenyl-4,8a-dimethyl-1,2,3,5,6,7,8,8a-octahydro-napthalene-2ol, megastigmatrienone, 4-dimethyamino-benzoic acid, 4-(dimethylamino)-,3,5-dimethyl-phenol, 6-isopropylidene-bicyclo[3.1.0]hexane, bicyclo[3.1.0]hexane-3one, 6-methyl-2(1h)-pteridinone, (1,1,4,4-tetramethyl-2,3-tetralindione), δ1(9)-2-octalone | Mukunthan et al. [102] |

**Table S1:** Continued.

| **Part of plant** | **Extraction / isolation methodology** | **Phytochemical detected** | **Reference** |
| --- | --- | --- | --- |
| Leaves | Isolation of essential oil by hydrodistillation | α-pinene, camphene, β-pinene, eucalyptol, L- linalool, verbenol, camphor, borneol, 1-borneol, trans-carveol, cis-carveol, α-fenchyl acetate, δ -elemene, β -elemene, trans-caryphyllene, β -farnesene, aromadendrene, α-humulene, murolene, germacrene-D, curzerene, junipene, nerolidol, germacrene- B, caryophyllene oxide, α-eudesmol, germacrone, curdione, confertin, neocyrdione, xanthinin, phytol | Borah et al. [42] |
| Leaves | Isolation of essential oil by hydrodistillation | α-pinene, β-pinene, myrcene, limonene, 1,8-cineole, terpinolene, camphor, linalool, β-elemene, β-caryophyllene, borneol, α-terpineol, methyl eugenol, (E)-methyl isoeugenol, farnesol | Behura and Srivastava  [113] |
| Rhizomes | Isolation of essential oil by hydrodistillation | Furanodiene, furanodienone, curzerenone and germacrone | Mahanta et al. [114] |
| Rhizomes | Isolation of essential oil by hydrodistillation | α-pinene, camphene, β-pinene, myrcene, δ-3-carene, p- cymene, limonene, eucalyptol, terpinolene, linalool, camphor, camphene hydrate, borneol, terpinene-4-ol, chavicol metyl, dihydrocarveol, acetate terpinene-4-ol, β-elemene, β-caryophyllene, ar-curcumene, germacrene D, β-selinene, zingiberene, δ- cadinene, elemol, germacrene B, caryophyllene oxide, Epi-globulol, ar-turmerone, β- eudesmol, α-cadinol, 7-Epi-α-eudesmol, germacrone, farnesol | Singh et al. [115] |


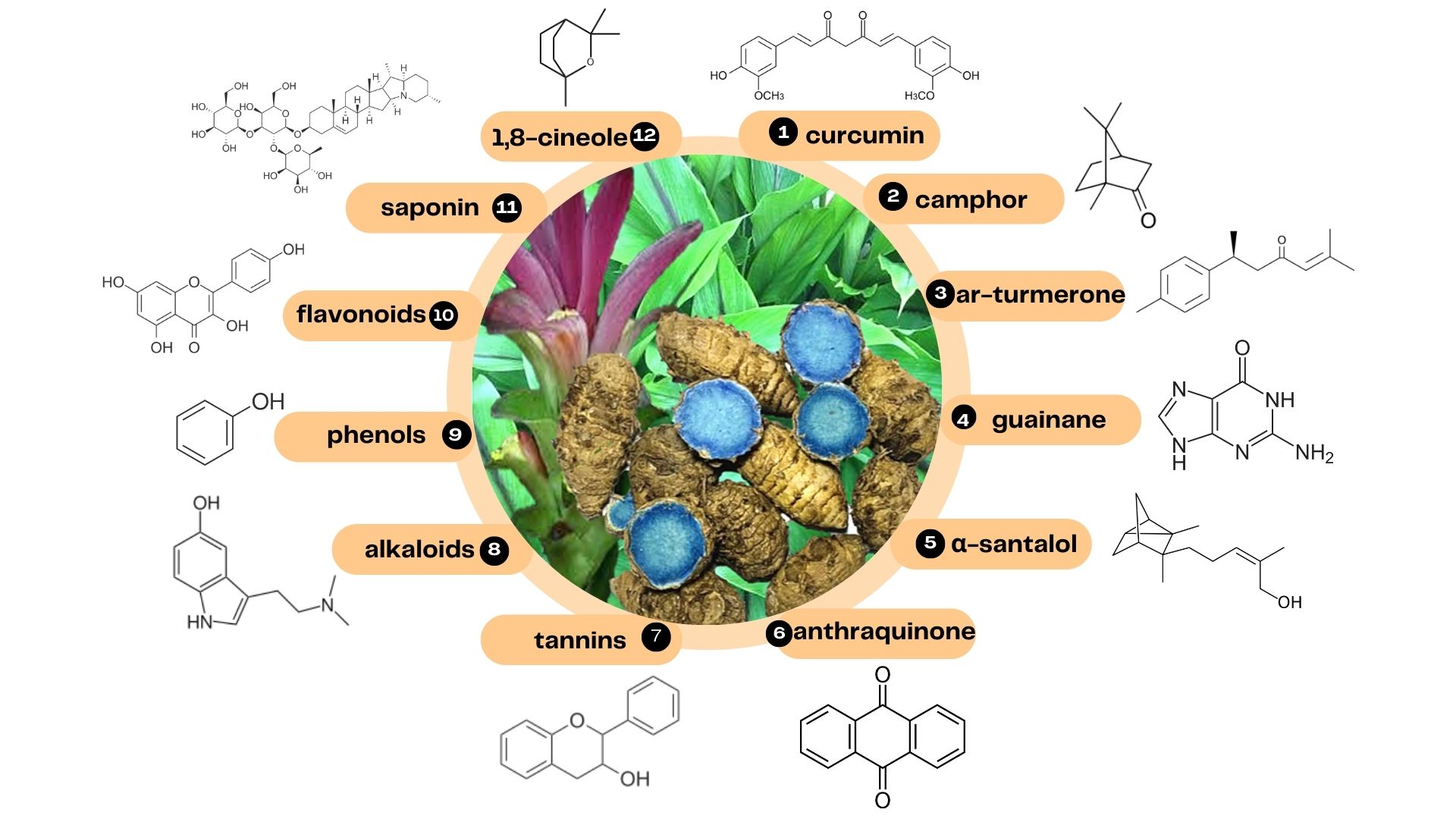


Figure S1: Phytochemical constituents of C. caesia
